# Assessing the Impact of SARS-CoV-2 Spike Mutations on Antibody Binding: A Comparative Assessment of the Wuhan and JN.1 Variants’ Full-Length Spikes in a Multiplex Luminex Assay

**DOI:** 10.3390/v17091248

**Published:** 2025-09-16

**Authors:** Gerald Waweru, Ruth Nyakundi, Bernadette Kutima, Sharon Owuor, Gloria Konyino, John Gitonga, Doreen Lugano, Angela Maina, Jennifer Musyoki, Lucy Ochola, Martin Omondi, Christopher K. Kariuki, Paul Ogongo, Christina Mwachari, Faiz Shee, Charles Agoti, Charles Sande, Sophie Uyoga, Eunice Kagucia, Ambrose Agweyu, Philip Bejon, J. Anthony G. Scott, George M. Warimwe, L. Isabella Ochola-Oyier, James Nyagwange

**Affiliations:** 1 KEMRI-Wellcome Trust Research Programme, Center for Geographic Medicine Research, Coast, Kilifi P.O. Box 230-80108, Kenya; 2 School of Pure and Applied Sciences, Department of Biological Sciences, Pwani University, Kilifi P.O. Box 195-80108, Kenya; 3 Kenya Institute of Primate Research, Nairobi P.O. Box 24481 00502, Kenya; 4 Kenya Medical Research Institute, Nairobi P.O. Box 54840 00200, Kenya; 5 Department of Infectious Diseases Epidemiology, London School of Hygiene and Tropical Medicine, London WC1E 7HT, UK; 6 Nuffield Department of Medicine, Oxford University, Oxford OX3 7BN, UK

**Keywords:** SARS-CoV-2, wildtype, omicron, ELISA, Luminex, sensitivity, specificity, agreement

## Abstract

Severe Acute Respiratory Syndrome Coronavirus 2 (SARS-CoV-2) continues to evolve, with mutations leading to the emergence of new variants. JN.1, a subvariant of omicron BA.2.86, has demonstrated marked immune escape and is now included in updated vaccine formulations. While reduced sensitivity has been reported for antibody assays using ancestral spike protein subunits to detect omicron-induced responses, the performance of full-length spike-based assays against omicron sublineages remains unclear. We aimed to compare the sensitivity of ELISA and Luminex assays using full-length spike proteins from the ancestral Wuhan strain and the JN.1 variant. Methods: Wuhan and JN.1 full-length spike protein constructs were designed and expressed in Expi293F mammalian cells. In-house ELISAs based on previously validated protocols were used to measure anti-spike IgG levels. Additionally, a Luminex-based assay for anti-spike antibody detection was developed and validated. Both assays were applied to the following sample groups: pre-pandemic samples (designated “gold standard negatives”); PCR confirmed 2020 positives (“gold standard wildtype positives”); PCR confirmed 2024 positives (“gold standard omicron positives”); 2022 vaccinated individuals with verbal confirmed infection (“gold standard hybrid positives”); and 2024 household samples (“unknowns”). Results: Wuhan spike protein showed a sensitivity of 100% (95% CI: 0.88–1.0) in detecting omicron-specific antibodies using gold standard omicron positives with JN.1 spike protein as a reference assay. Overall, across all samples, in ELISA, the Wuhan antigen had a sensitivity of 0.93 (95% CI: 0.89–0.95) and a specificity of 0.98 (95% CI: 0.94–0.99). The JN.1 antigen showed a sensitivity of 0.91 (95% CI: 0.87–0.94) and a specificity of 0.97 (95% CI: 0.93–0.99). In Luminex, sensitivity was 0.95 (95% CI: 0.91–0.97) for Wuhan and 0.94 (95% CI: 0.91–0.96) for JN.1. Specificity for both antigens in Luminex was 0.98 (95% CI: 0.94–0.99). Conclusions: Both ELISA and Luminex assays showed comparable sensitivity and specificity for both Wuhan and JN.1 antigens, indicating that mutations in the JN.1 variant do not significantly impact assay performance. This suggests preserved antigenic recognition across variants.

## 1. Introduction

SARS-CoV-2, the virus that causes coronavirus disease 2019 (COVID-19), first emerged in late 2019. It rapidly spread worldwide and the World Health Organization (WHO) declared the COVID-19 pandemic in March 2020. Following the virus’ emergence, various serological assays were developed to detect SARS-CoV-2-specific antibodies [1,2,3]. These assays utilize different capture antigens, including the nucleocapsid (N) protein, the whole spike (S) protein, or specific segments of the spike protein, such as the receptor-binding domain (RBD) [4,5,6]. Spike protein-based assays are generally preferred due to the protein’s high immunogenicity, which elicits strong and measurable antibody responses [7]. These assays have played a crucial role in SARS-CoV-2 epidemiological studies, providing insights into population exposure and vaccine-induced immunity [8].

The SARS-CoV-2 spike protein is made up of two domains, the S1 domain and the S2 domain [9]. Genetic studies have shown that the S1 domain is more variable with mutations occurring mainly within its RBD, while the S2 domain is more conserved with fewer mutations [10]. Due to these mutations and the immune pressure exerted on the spike protein by the host, different variants of the virus have been reported [11]. The omicron variant, currently the dominant circulating strain, harbors the highest number of mutations within the RBD, enhancing its ability to evade host immune responses [12]. In addition, escape mutations have also been reported within the N-terminal domain (NTD) of the omicron spike protein, which are associated with an increased rate of immune evasion [13,14]. Multiple studies have reported that omicron subvariants have decreased sensitivity to neutralization by antibodies raised from exposure to earlier subvariants or by vaccination [15,16,17,18,19,20]. In Kenya, Lugano et al. (2024) demonstrated that antibodies from the general population, sampled in 2022 and 2023, were less effective in neutralizing omicron subvariants such as FY.4, BA.2.86, JN.1, JN.1.4, and KP.3.1.1 compared to the ancestral virus [19]. The extensive mutations in omicron have also been reported to weaken the binding of monoclonal antibodies (mAbs), allowing the omicron variants to escape neutralization by approved COVID-19 therapeutic antibodies [21].

Previous studies have demonstrated that antibody assays using wildtype virus S1 and RBD as capture antigens have reduced sensitivity for detecting antibodies elicited by omicron exposure [22]. This observation raises the question of whether mutations in the omicron spike protein similarly compromise the sensitivity of assays that use the wildtype virus’s full-length spike as the capture antigen.

This study aimed at comparing a well-validated in-house ELISA based on the Wuhan full-length spike with the JN.1 full-length spike assay. Selection of the JN.1 full-length spike followed the World Health Organization’s Strategic Advisory Group of Experts on Immunization’s (SAGE) recommendation of updating COVID-19 vaccines to include a monovalent JN.1 spike antigen in April 2024 [23]. Therefore, the JN.1 spike assay would additionally be useful in monitoring JN.1 vaccine-induced antibodies.

## 2. Materials and Methods

### 2.1. Sample Sets and Ethical Considerations

The characteristics of the test samples are shown in Table 1. The set of gold standard negative samples comprised 160 pre-pandemic serum samples collected in 2018 as part of research into the quality of transfused blood in coastal Kenya [24,25]. The wildtype gold standard positive samples consisted of serum from 160 COVID-19 patients collected ≥7 days and <120 days after a positive PCR diagnosis in 2020 [24,25]. Additionally, there were hybrid positive samples from the Nairobi Health and Demographic Surveillance System (HDSS) which consisted of 160 samples from infected and vaccinated individuals. Vaccination was confirmed through a vaccination certificate or short message service message (SMS), sent to their phones after vaccination, while infection was defined as verbal report of previous exposure to the virus. The median number of days post vaccination was 271 (IQR = 200, 324 days). These samples were collected in 2022 during the COVID-19 pandemic [26]. Omicron gold standard positive samples included 32 omicron PCR-confirmed samples collected in Kilifi HDSS, with samples collected between two and three months post infection. Finally, 400 household samples collected in Kilifi HDSS in late 2023 and early 2024 designated as unknowns were included in the assays.

Ethical approval for the collection and use of these samples was provided by the Scientific and Ethical Review Unit (SERU) at the Kenya Medical Research Institute, number and initial protocol approval date KEMRI/SERU/CGMR-C/203/4085 (26 August 2020), 1433 (3 November 2008), 3426 (31 March 2017), 4724 (4 July 2025), and 3149 (15 January 2016); the Oxford Tropical Research Ethics Committee (44-20); and the London School of Hygiene and Tropical Medicine Research Ethics Committee (26950). The participants signed consent to have their blood collected and used for future research purposes.

### 2.2. Construct Design and Protein Expression

Protein expression was performed as previously described [24]. Briefly, constructs for both Wuhan and JN.1 spike proteins were designed and optimized for mammalian expression. The constructs were transformed into competent One Shot™ TOP10 *E. coli* cells (cat no. C404003, ThermoFisherScientific, Waltham, MA, USA) for plasmid amplification, and the plasmids were purified using a Maxiprep plasmid purification kit according to the manufacturer’s protocol (cat no. 12263, QIAGEN™, Hilden, Germany).

Protein expression was carried out using the ExpiFectamine™ 293 Transfection Kit (cat no. A14525, ThermoFisherScientific, Waltham, MA, USA), following the manufacturer’s instructions. Purification was performed by affinity binding using ProBond™ resin (cat no. 46-0019, ThermoFisherScientific, Waltham, MA, USA), as per the manufacturer’s protocol.

### 2.3. ELISA Assays

The ELISA assay was performed following the in-house SARS-CoV-2 ELISA protocol established previously at KWTRP [24,25]. Briefly, Nunc MaxiSorp™ 96-well plates (cat no. 442404, ThermoFisherScientific, Waltham, MA, USA) were coated with 2 µg/mL of either wildtype or JN.1 trimeric spike protein in phosphate-buffered saline (PBS) and incubated at 37 °C for 1 h. Plates were then washed with wash buffer (0.1% Tween 20 in 1X PBS) and blocked with Blocker™ Casein (cat no. 37528, ThermoFisherScientific, Waltham, MA, USA) for 1 h at room temperature to minimize nonspecific binding. Plasma samples, diluted at 1:800 in Blocker™ Casein, were added and incubated for 2 h at room temperature. After washing, Horseradish Peroxidase (HRP)-conjugated goat anti-human IgG (cat no. 074-1002, KPL-SeraCare, Milford, MA, USA) was added and incubated for 1 h, followed by substrate development using o-phenylenediamine dihydrochloride (OPD) substrate (cat no. P8412, Sigma St. Louis, MO, USA). The reaction was stopped with 3M hydrochloric acid (HCl), and absorbance was measured at 492 nm. The results were expressed as OD (optical density) ratios, calculated by dividing each sample OD with the average OD of negative controls in the corresponding plate. Samples were classified as seropositive if the OD ratio ≥ 2 and seronegative if <2. Antibody concentrations, BAU/mL, were estimated with reference to the WHO International Standard for anti-SARS-CoV-2 immunoglobulin, NIBSC 20/136, as previously described [26]. Briefly, the sample OD ratio was divided by the OD ratio of the reference standard and the quotient multiplied by 1000.

### 2.4. Luminex Assay

Luminex MagPlex^®^ Microspheres (xMAP^®^ Reagents, Luminex Corporation, Austin, TX, USA) were conjugated with JN.1 and wildtype spike proteins following the manufacturer’s protocol. Briefly, regions 38 and 42 of the MagPlex^®^ Microspheres were conjugated with the JN.1 and wildtype spike proteins, respectively. Then, 1 mL of the bead stock was transferred into a 1.5 mL Eppendorf tube and washed with 250 μL of distilled water. The beads were vortexed and sonicated for 20 s and placed in a magnetic separator for 60 s to remove the supernatant. Activation was carried out by adding 100 μL of activation buffer (0.1M sodium phosphate monobasic, pH 6.2) and mixing through vortexing and sonication. After removing the supernatant, 10 μL of 50 mg/mL sulfo-NHS (cat no. 24510, Thermoscientific, Waltham, MA, USA) and 10 μL of 50 mg/mL EDC (cat no. 22980, Thermoscientific, Waltham, MA, USA) were added. The mixture was incubated at room temperature for 20 min with vortexing at 10 min intervals. Following activation, the supernatant was removed, and the beads were washed three times with 250 μL of 50 mM MES buffer (pH 5.0). For protein conjugation, 50 μg of either JN.1 or wildtype spike protein was used to conjugate 1 mL of beads, and the volume was adjusted with MES buffer to reach a final volume of 1mL. The mixture was incubated for two hours at room temperature while shaking. After incubation, the supernatant was removed, and the beads were washed three times with 250 μL of wash buffer containing 1X PBS, 0.05% Tween-20, 1% BSA, and 0.1% sodium azide. The conjugated beads were then stored in 1 mL of 1X PBS, protected from direct light.

For the Luminex assay, the conjugated beads were incubated with samples diluted at 1:100 in 1X PBS-TBN (0.05% Tween-20, 1% BSA, and 0.1% sodium azide). The incubation was carried out for 45 min in a 96-well flat-bottom microplate (cat no. 780261, Greiner Bio-One International, Kremsmunster, Austria), using 90,000 beads per plate. The plates were then washed three times with wash buffer. After washing, a secondary antibody, R-Phycoerythrin AffiniPure™ Goat Anti-Human IgG, Fcγ fragment specific (cat no. 109-116-097, Jackson ImmunoResearch, West Grove, PA, USA), was added at a 1:400 dilution in 1X PBS. The plates were incubated at room temperature for 30 min while shaking, after which they were washed four times with wash buffer. After the final wash, 100 μL of 1X PBS was added to each well, and the plates were analyzed using a Luminex analyzer (LX 200 machine, Luminex Corporation, Austin, TX, USA). The results were expressed as net mean fluorescence intensity (MFI) ratios, calculated by dividing each sample MFI with the average MFI of negative controls in the corresponding plate. Samples were classified as seropositive if the MFI ratio ≥ 2 and seronegative if <2. Antibody concentrations, BAU/mL, were estimated with reference to the WHO International Standard for anti-SARS-CoV-2 immunoglobulin, as previously described [26]. Briefly, the sample MFI ratio was divided by the MFI ratio of the reference standard, NIBSC 20/136, and the quotient multiplied by 1000.

### 2.5. Data Analysis

Data analysis was conducted using R version 4.2.2. Correlation analysis was performed to assess the relationship between responses to the two antigens in both ELISA and Luminex assays. The Wilcoxon signed-rank test was used to determine significant differences between OD ratios, MFI ratios, and BAU/mL for the two antigens. Cohen’s kappa test was applied to evaluate the agreement between the two antigens in serostatus classification. To assess the potential loss of sensitivity of the Wuhan-based assay in detecting JN.1-induced antibodies, sensitivity was calculated using the JN.1 antigen as the reference standard. Exact (Clopper–Pearson) 95% confidence intervals were computed. Receiver operating characteristic (ROC) curves were generated using gold standard negative and positive samples to assess whether differences between the assays were intrinsic to the assay or influenced by the selected cutoffs. Assay reproducibility was assessed by examining raw OD and MFI values and calculating the coefficient of variation (CV) for positive and negative controls.

## 3. Results

We used the JN.1 ELISA and Luminex assays as references to assess the loss of sensitivity in the Wuhan ELISA and Luminex assays for detecting antibodies against the omicron variant (Table 2). In both assays, the Wuhan antigen classified all omicron samples correctly, demonstrating a sensitivity of 100% (95% CI: 88-100) (Table 2).

Overall, across all sample groups, in ELISA, the Wuhan antigen showed a sensitivity of 0.93 (95% CI: 0.89–0.95) and a specificity of 0.98 (95% CI: 0.94–0.99), while JN.1 had a sensitivity of 0.91 (95% CI: 0.87–0.94) and a specificity of 0.97 (95% CI: 0.93–0.99). In Luminex, sensitivity was higher for both antigens, 0.95 (95% CI: 0.91–0.97) for Wuhan and 0.94 (95% CI: 0.91–0.96) for JN.1, with both showing a specificity of 0.98 (95% CI: 0.94–0.99).

We then assessed the agreement in serostatus classification between the Wuhan and JN.1 spike antigens using Cohen’s kappa statistic. Agreement between the Wuhan and JN.1 ELISA assays was high, with a kappa coefficient of 0.94, indicating almost perfect agreement. Similarly, the Luminex assays showed a kappa of 0.98 between the Wuhan and JN.1 antigens. Cross-platform agreement was also strong: the kappa for Wuhan ELISA vs. Wuhan Luminex was 0.91, and for JN.1 ELISA vs. JN.1 Luminex, 0.88—both reflecting near-perfect consistency in serostatus classification across platforms (Table 3).

**Figure 1:**
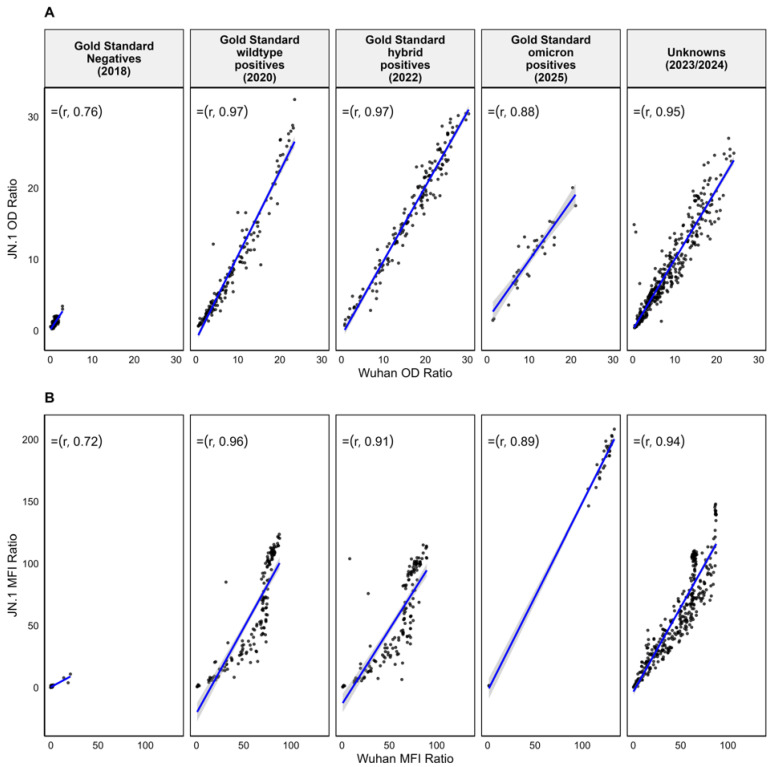
Correlation between Wuhan and JN.1 OD ratios and MFI ratios across different samples. Scatter plots showing the correlation between Wuhan and JN.1 OD ratios (**A**) and MFI ratios (**B**) across five sample groups. Each plot includes a fitted regression line (blue) and Spearman correlation coefficient.

We compared the correlation of the OD and MFI ratio between the Wuhan and JN.1 antigens across all sample groups (Figure 1). In the gold standard negatives, the correlation was ρ = 0.76 (95% CI [0.667–0.826]). The gold standard wildtype positives showed a correlation of ρ = 0.97 (95% CI [0.956–0.984]), gold standard hybrid positives had a correlation of ρ = 0.97 (95% CI [0.952–0.981]), while in gold standard omicron positives the correlation was ρ = 0.88 (95% CI [0.85–0.91]). In the unknowns, the correlation was ρ = 0.95 (95% CI [0.920–0.973]) (Figure 1A).

A strong correlation was observed between the Wuhan and JN.1 MFI ratios across all sample groups (Figure 1B). In the gold standard negatives, the correlation was ρ = 0.72 (95% CI [0.609–0.808]). In the gold standard wildtype positives, the correlation was ρ = 0.96 (95% CI [0.927–0.974]) while the correlation in the gold standard hybrid positives was ρ = 0.91 (95% CI [0.85–0.941]). Similarly, the correlation in gold standard omicron positives was ρ = 0.89 (95% CI [0.85–0.91]), while in the unknowns the correlation was ρ = 0.94 (95% CI [0.92–0.973]) (Figure 1B).

**Figure 2:**
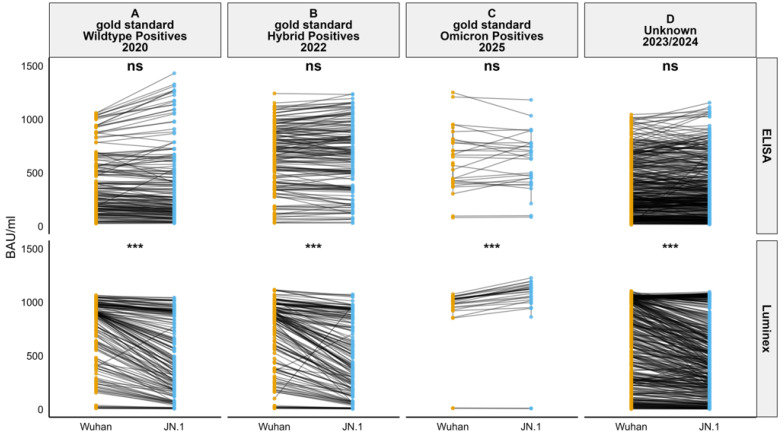
A paired comparison of BAU/mL for JN.1 (sky blue) and Wuhan (orange) antigens in ELISA and Luminex assays across sample groups: gold standard wildtype positives (**A**), gold standard hybrid positives (**B**), gold standard omicron positives (**C**), and unknowns (**D**). Slope plots show paired values, with the Wilcoxon test used for significance. “***” indicates *p* < 0.001, “ns” indicates no significance.

Antibody concentrations were calculated across all sample groups. The Wilcoxon test showed no significant difference in BAU/mL units across sample groups when measured using ELISA (*p* > 0.05). However, a significant difference was observed in the gold standard wildtype positives, gold standard hybrid positives, gold standard omicron positives, and unknown sample groups when using Luminex (*p* < 0.001). A paired comparison for each individual sample between the Wuhan and JN.1 BAU/mL values in gold standard wildtype positives, gold standard hybrid positives, gold standard omicron positives, and unknown samples was carried out using slope plots (Figure 2).

**Figure 3:**
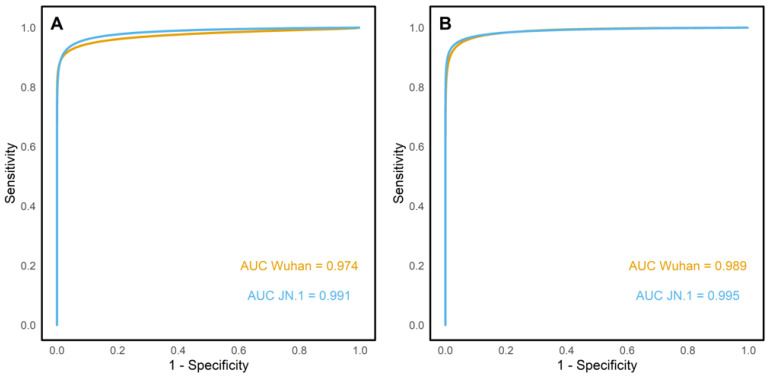
ELISA (**A**) and Luminex (**B**) ROC curves. It shows high accuracy for both assays: ELISA AUCs were 0.974 (Wuhan) and 0.991 (JN.1); Luminex AUCs were 0.987 (Wuhan) and 0.994 (JN.1).

Receiver operating characteristic (ROC) curves were plotted for Wuhan ELISA and JN.1 ELISA (Figure 3). For the Wuhan antigen, the ROC curves were plotted using the gold standard negatives, the gold standard wildtype positives, and the gold standard hybrid positives. Similarly, JN.1 ROC curves were plotted using the gold standard negatives and the gold standard omicron positives samples. In the ELISA assay, the Wuhan and JN.1 antigens had an area under the curve (AUC) of 0.974 and 0.991, respectively. Similarly, the Luminex ROC curve had an AUC of 0.989 for the Wuhan antigen and 0.995 for the JN.1 antigen.

## 4. Discussion

This study aimed at determining the effect of omicron spike mutations on the sensitivity of binding assays utilizing full-length spike proteins. Rössler et al. (2023) reported reduced sensitivity of commercial assays utilizing S1 and RBD segments of the spike protein in detecting binding antibodies following omicron infection [27]. However, the impact of the full-length spike mutations on the sensitivity of binding assays has not been reported and it is generally assumed that the responses against the conserved S2 domain would cumulatively result in sustained sensitivity of these assays [28,29,30]. To formally evaluate the impact of the mutations on full-length protein assay sensitivity, we compared ELISA responses and Luminex assay responses using JN.1 and Wuhan full-length spike antigens. There was no loss of sensitivity of the Wuhan spike in detecting omicron-specific antibodies when compared to the JN.1 spike, with both showing sensitivity of 100% (95% CI: 88–100). Furthermore, both JN.1 and Wuhan spike antigens had comparable sensitivity and specificity in both the ELISA and Luminex assays across sample sets collected during the periods of Wuhan and omicron circulation as well as post vaccination [24,25,26]. These results reinforce the usefulness of the conserved S2 domain in sustaining the sensitivity of the full-length spike assays [28,29,30].

A comparison of antibody concentrations (BAU/mL) revealed no significant differences in the quantity of binding antibodies detected using ELISA for the two antigens. These results were consistent with those reported by Hussain et al. (2025), which showed no significant difference in binding antibodies detected by omicron variants such as BA.1, BA.2, BA.4/5, BQ.1.1, and XBB.1.5 in ELISA [31]. However, in the Luminex assay, a significant difference in the BAU/mL was observed between the Wuhan and JN.1 antigens. This difference may be attributed to how antigens are presented in each assay format. On the Luminex platform, spike proteins are covalently bound to beads in suspension, which may better preserve conformational epitopes and facilitate multivalent antibody binding. In contrast, ELISA involves passive adsorption of antigen onto plastic surfaces, which can alter protein conformation and limit epitope accessibility. These differences in antigen presentation, combined with the broader dynamic range and multiplex capability of Luminex, may contribute to its increased sensitivity in detecting variant-specific antibody responses. In gold standard wildtype positives, gold standard hybrid positives, and unknowns, the antibody binding to the Wuhan spike protein was consistently higher than that to the JN.1 spike protein. These results align with the findings of Kuthning et al. (2024), which reported stronger wildtype-based antibody binding to the S1 domain of Wuhan and alpha variants compared to the S1 domain of delta, omicron BA.1, BA.2, and BA.4/5 variants [32]. This pattern was expected, as the gold standard wildtype positives consisted of sera collected in 2020, primarily containing antibodies against the ancestral Wuhan strain [24,25]. Similarly, the gold standard hybrid positives consist of sera from individuals vaccinated with Wuhan spike-based vaccines [26]. As expected, in the gold standard omicron positives, the antibody binding units to the omicron JN.1 antigen were higher than to the Wuhan antigen, consistent with a high homologous response following omicron infection.

Serostatus agreement analysis supported the reliability of both spike-based ELISA and Luminex assays in detecting binding antibodies against the SARS-CoV-2 spike protein. Both intra-assay and inter-assay comparisons yielded an almost perfect agreement. These findings were reinforced by a ROC curve analysis which yielded high AUC values for both antigens; thus, both antigens had high discriminatory performance when classifying positive and negative samples. Interestingly, the high agreement in serostatus classification observed in this study contrasts with reports by Springer et al. (2022), who documented that antigenic changes in the omicron variant significantly affect the diagnostic ability of commercial ELISA kits using S1 domain and RBD as the capture antigen [22]. These findings support the robustness of full-length spike protein-based assays, with both ELISA and Luminex demonstrating reliable detection of binding antibodies across SARS-CoV-2 variants. Despite minor differences in binding antibody concentrations, BAU/mL—particularly in the Luminex assay—the sensitivity and specificity of both platforms remained comparable, indicating that omicron spike mutations do not significantly affect assay performance. Together, the results underscore the continued utility of such assays in serological studies, even as new variants emerge.

## Figures and Tables

**Table 1:** Characteristics of the study population and sample classification.

Population	N	Date	Location	Participant Group	Timeline	Designation
Adult blood donors	160	2018	Coastal Kenya	Adults investigated for blood transfusion safety	No previous SARS-CoV-2 infection	Gold standard negatives
SARS-CoV-2 PCR-positive cohort	160	2020	Nairobi	Adults with wildtype confirmed sequences	Collected ≥7 days and <120 days after PCR positive diagnosis	Gold standard wildtype positives
Nairobi HDSS vaccinated	160	2022	Nairobi	Adults and children investigated for SARS-CoV-2 seroprevalence	Median 271 days post vaccination; previous exposure possible but timing unknown	Gold standard hybrid positives
Kilifi HDSS	32	2025	Kilifi	Adults with omicron-confirmed sequences	Collected ≤3 months post infection	Gold standard omicron positives
Kilifi household samples	400	2023/24	Kilifi	Adults and children investigated for respiratory viruses’ reinfection	No records for infection or vaccination	Unknowns

**Table 2:** Comparison between JN.1 and Wuhan spikes in classifying omicron gold standard positives by both ELISA and Luminex.

	Wuhan Luminex/ELISA			
		Negative	Positive	Total
**JN.1 Luminex/ELISA**	Negative	2	0	2
	Positive	0	30	30
	**Total**	2	30	32

**Table 3:** Agreement between ELISA and Luminex assays for Wuhan and JN.1 spike antigens using all gold standard samples.

		Negatives	Positives	Total	Cohen’s Kappa
		**JN.1 ELISA**			
**Wuhan ELISA**	Negatives	178 (35%)	4 (0.8%)	182 (35.8%)	0.94
	Positives	9 (1.8%)	321 (62.7%)	330 (64.2%)	
	Total	187 (36.8%)	325 (63.5%)	512 (100%)	
		**JN.1 Luminex**			
**Wuhan Luminex**	Negatives	174 (34%)	1 (0.2%)	175 (34.2%)	0.98
	Positives	3 (0.6%)	334 (65.2%)	337 (65.8%)	
	Total	177 (34.6%)	335 (65.4%)	512 (100%)	
		**Wuhan Luminex**			
**Wuhan ELISA**	Negatives	168 (32.8%)	14 (2.7%)	182 (35.5%)	0.91
	Positives	7 (1.4%)	323 (63.1%)	330 (64.5%)	
	Total	175 (34.2%)	337 (65.8%)	512 (100%)	
		**JN.1 Luminex**			
**JN.1 ELISA**	Negatives	168 (32.8%)	19 (3.7%)	187 (36.5%)	0.88
	Positives	9 (1.8%)	316 (61.7%)	325 (63.5%)	
	Total	177 (38.5%)	335 (61.5%)	512 (100%)	

## Data Availability

Deidentified data can be accessed in the Harvard Dataverse via the link DOI: https://doi.org/10.7910/DVN/T0Q22I.

## Abbreviations

MFI	Mean Fluorescence Intensity
ELISA	Enzyme-Linked Immunosorbent Assay
OD	Optical Density
ROC	Receiver Operating Characteristic
BAU	Binding Antibody Units
